# Study of Effective Stress Intensity Factor through the CJP Model Using Full-Field Experimental Data

**DOI:** 10.3390/ma16165705

**Published:** 2023-08-20

**Authors:** Alonso Camacho-Reyes, Jose Manuel Vasco-Olmo, Giancarlo Luis Gómez Gonzales, Francisco Alberto Diaz

**Affiliations:** Departamento de Ingeniería Mecánica y Minera, Universidad de Jaén, 23071 Jaen, Spain; jvasco@ujaen.es (J.M.V.-O.); glgomez@ujaen.es (G.L.G.G.); fdiaz@ujaen.es (F.A.D.)

**Keywords:** fatigue crack growth, CJP model, crack tip parameters, crack tip shielding, digital image correlation, thermoelastic stress analysis

## Abstract

In this work, the Christopher–James–Patterson crack tip field model is used to infer and assess the effective stress intensity factor ranges measured from thermoelastic and digital image correlation data. The effective stress intensity factor range obtained via the Christopher–James–Patterson model, which provides an effective rationalization of fatigue crack growth rates, is separated into two components representing the elastic and retardation components to assess shielding phenomena on growing fatigue cracks. For this analysis, fatigue crack growth tests were performed on Compact-Tension specimens manufactured in pure grade 2 titanium for different stress ratio levels, and digital image correlation and thermoelastic measurements were made for different crack lengths. A good agreement (~2% average deviation) was found between the results obtained via thermoelastic stress analysis and digital image correlation indicating the validity of the Christopher–James–Patterson model to investigate phenomena in fracture mechanics where plasticity plays an important role. The results show the importance of considering crack-shielding effects using the Christopher–James–Patterson model beyond considering an exclusive crack closure influence.

## 1. Introduction

The CJP model (Christopher, James, and Patterson) [1,2,3,4] is a crack tip field model able to reproduce the effect of the plastic enclave surrounding a fatigue crack on the elastic field. Therefore, this model is able to quantify the crack-shielding effect [5,6,7] during fatigue crack growth (either crack growth acceleration or retardation). Unlike other elastoplastic based models such as the HRR fields [8], the CJP model approach is different since it is mathematically based on Muskhelishvili’s complex potentials [9] (linear and elastic). Beyond the extensive experience of its authors in several research fields, the development of the CJP model is a result of the evolution of full-field optical techniques [10,11,12] that have been evidenced as highly capable in quantifying the effective driving force for crack growth through the experimental measurement of crack tip displacement or stress fields [13,14,15,16,17]. The CJP model was originally developed and validated using Digital Photoelasticity [1,2], subsequently extended to Digital Image Correlation (DIC) for its application to metallic materials [3,4], and recently validated for its use in Thermoelastic Stress Analysis (TSA) [18]. Many works, which will be described below, can be found in the literature and show the ability of the model for reproducing crack-shielding effects. Examples are the works conducted by Vasco-Olmo et al. [19] and Robles et al. [20] showing the ability of the model to detect the crack closure/opening loads throughout a fatigue loading cycle; the work reported by Vasco-Olmo et al. [21] showing the ability of the model to accurately reproduce the size and shape of the crack tip plastic zone; the works by Nowell et al. [22,23] showing the agreement of the model with a displacement partitioning elastic–plastic model [24]; and Yang et al.’s work [25] showing how the CJP model effective stress intensity factor range can rationalize fatigue crack growth rates for different specimens and stress ratios.

As will be described in Section 2, the CJP model describes the crack tip stress field through several coefficients that define three stress intensity factors accounting for crack plasticity effect. In previous described works, authors used these stress intensity factors to analyze crack-shielding effect. Although has been proved that the CJP model is effective in terms of modelling crack tip plasticity effects on the elastic field as previously described [21,23], sometimes the role of each parameter in modelling the crack-shielding effect is not entirely obvious. This is due to the coefficient that is related to crack growth retardation (or acceleration) since it appears in the two stress intensity factors that define the effective stress intensity range. Thus, in this work, an analysis of the ranges of the CJP model coefficients experimentally calculated via TSA and DIC is performed in order to investigate the role of each parameter. The aim is to define and compare both the elastic and retardation parts of the CJP model effective stress intensity factor range regarding nominal values and their influence on fatigue crack growth rates.

## 2. Mathematical Description of the Crack Tip Fields According to the CJP Model

The CJP model is based on Nurse and Patterson’s approach [26] for the description of Muskhelishvili’s complex potentials [9] as Fourier series. The stress tensor components surrounding a fatigue crack according to this model are given by Equations (1)–(3) [1]:

σ_x_ = r^−1/2^ {−1/2 (A + 4B + 8E) cos(θ/2) − 1/2 B cos(5θ/2) − 1/2 E [ln(r) (cos(5θ/2) + 3 cos(θ/2)) + θ (sin(5θ/2)+ 3 sin(θ/2))]} + C, (1)

σ_y_ = r^−1/2^ {1/2 (A − 4B − 8E) cos(θ/2) + 1/2 B cos(5θ/2) + 1/2 E [ln(r) (cos(5θ/2) − 5 cos(θ/2)) + θ (sin(5θ/2) − 5 sin(θ/2))]} − F, (2)

**Figure 1:**
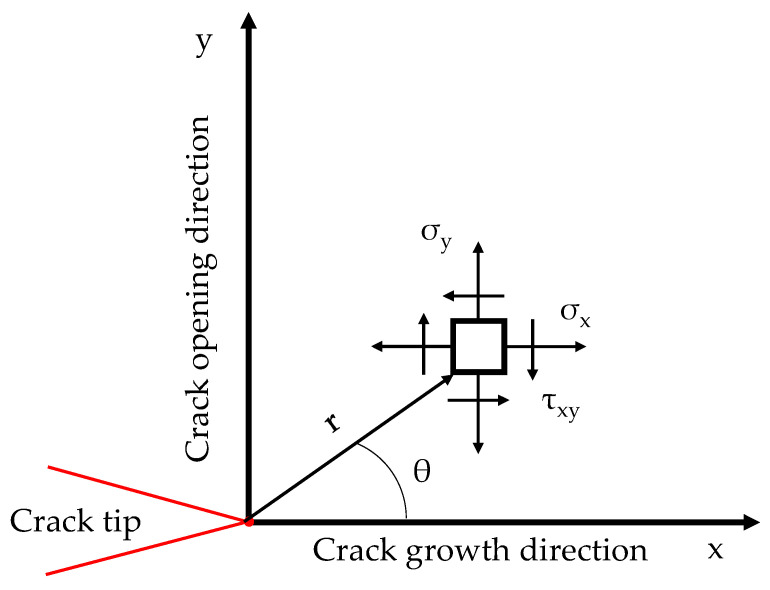
Stress tensor definition at a point surrounding the crack tip in terms of polar coordinates [27].

τ_xy_ = r^−1/2^ {A sin(θ/2) + B sin(5θ/2) − E sin(θ) (ln(r) cos(3θ/2) + θ sin(3θ/2))}, (3)

where r and θ are the polar coordinates in the crack plane (see Figure 1), and A, B, C, E, and F are the coefficients describing the stress fields. Those coefficients provide three stress intensity factors and two non-singular stresses along the crack growth and opening directions. The first stress intensity factor is K_F_, which is assumed as the driving force forward the crack tip. In other words, K_F_ is an equivalent mode I stress intensity factor that takes into account crack-shielding effects. K_F_ is obtained by combining the coefficients as follows:

(4) $$K_{F}=\sqrt{\frac{\pi }{2}}\;\left(A-3B-8E\right),$$

The second SIF, which is defined as the retardation stress intensity factor, is denoted by K_R_ and represents the retardation or acceleration due to the crack-shielding effect. This SIF is directly obtained through coefficient E as shown in Equation (5):

(5) $$K_{R}=-4\pi \sqrt{\frac{\pi }{2}}E,$$

The third stress intensity factor is the compatibility-induced shear stress intensity factor, which is denoted by K_S_. It characterizes the shear stress on the elastoplastic boundary. K_S_ is given by Equation (6):

(6) $$K_{S}=\sqrt{\frac{\pi }{2}}\left(A+B\right),$$

Finally, the coefficients C and F are related to non-singular stresses along the crack growth and the crack opening directions, respectively:

σ_0x_ = −C (7)

σ_0y_ = −F (8)

where σ_0x_ and σ_0y_ are the non-singular stresses along the crack growth and crack opening directions, respectively.

The effective stress intensity factor according to the CJP model is obtained by combining K_F_ and K_R_ [25]. Equation (9) gives the ΔK_eff_ as a function of the CJP model coefficients:

(9) $${\Delta K}_{\mathrm{eff}}=\sqrt{\frac{\pi }{2}}\left(A-3B+4.56E\right),$$

The crack tip displacement fields are given by:

u_x_ = 1/(2G) {r^1/2^ (−A − 2Bϰ − 2E) cos(θ/2) + r^1/2^ (B + 2E) cos(θ/2) − Er^1/2^ [ln(r) (cos(3θ/2) + (1 − 2ϰ) cos(θ/2)) + θ (sin(3θ/2) + (2ϰ − 1) sin(θ/2))] − C r (1 + ϰ) cos(θ)/4 + F r (ϰ − 3) cos(θ)/4}, (10)

u_y_ = 1/(2G) {r^1/2^ (A − 2Bϰ − 2E) cos(θ/2) + r^1/2^ (B + 2E) cos(θ/2) + Er^1/2^ [ln(r) (sin(3θ/2) − (1 + 2ϰ) sin(θ/2)) − θ (cos(3θ/2) + (2ϰ + 1) cos(θ/2))] + C r (3 − ϰ) sin(θ)/4 + F r (ϰ + 1) sin(θ)/4}, (11)

In the above equations, ϰ is (3–4 ν) for plane strain and (3 − ν)/(1 + ν) for plane stress. G and ν are the shear modulus and Poisson’s ratio, respectively.

## 3. Experimental Campaign

**Figure 2:**
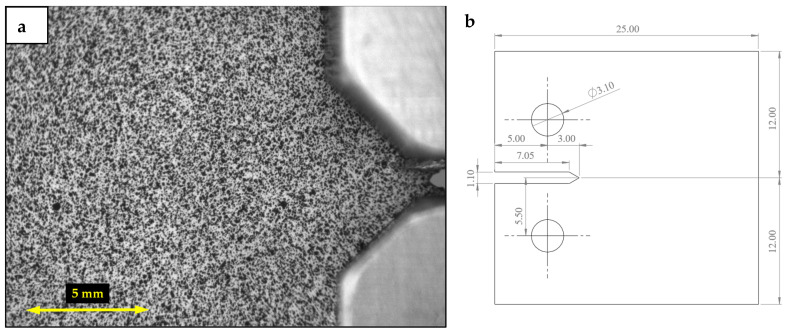
CT specimen (1 mm thickness). (**a**) DIC image of the specimen at the beginning of the test (undamaged) and (**b**) drawing indicating the specimen dimensions (mm).

Fatigue crack growth tests were performed on a servo hydraulic machine testing (MTS 370.02) on Compact-Tension specimens manufactured in pure grade 2 titanium. Table 1 and Table 2 show the chemical composition and the mechanical properties, respectively, and Figure 2 shows the employed CT specimen both in a DIC image and in a drawing showing its dimensions. Two stress ratios (0.1 and 0.6) were tested holding the maximum load at 750 N with a loading frequency of 11 Hz. The 2D-DIC and TSA techniques were employed for the simultaneous measurement of displacement fields and thermoelastic data, respectively. The specimens were previously prepared for TSA and DIC measurements by applying a black matte paint layer on the front specimen’s side to increase the thermal emissivity for TSA measurements and spraying a stochastic black and white speckle pattern on the backside of the specimen for correct DIC processing.

**Figure 3:**
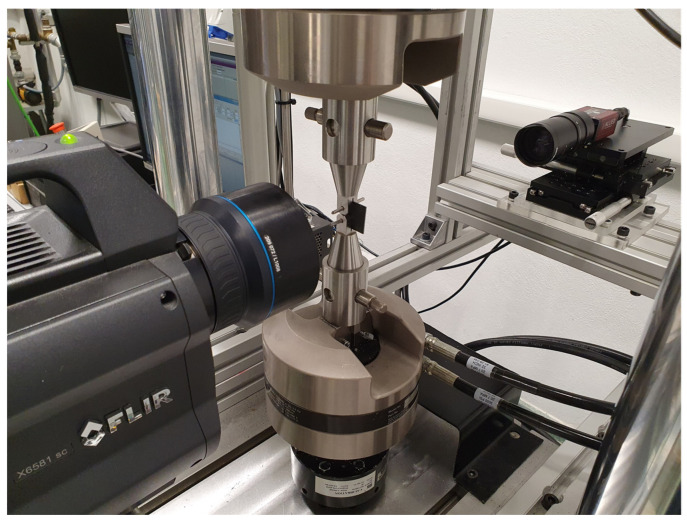
Experimental setup for fatigue testing and DIC and TSA data acquisition.

TSA and DIC measurements were performed for different crack lengths approximately within the range between 5 and 10 mm (0.25 and 0.5 in terms of the dimensionless crack length). For TSA measurements, a cooled infrared camera (model FLIR X6581 SC, Teledyne FLIR Spain, Alcobendas, Madrid) was employed as shown in Figure 3. A 50 mm lens along with an extension ring were employed to increase the spatial resolution. Thus, a spatial resolution of 35.1 μm per pixel was obtained. The acquisition time was 2 s, and the acquisition frame rate was 300 Hz. Thus, 22 cycles with 27 points per cycle were acquired. An integration time of 2 ms was chosen. From the measured temperature maps, the phase and amplitude temperature maps were calculated by fitting temperature–time data to Fourier series as reported in reference [18].

For 2D-DIC data acquisition, a 2 MPixels Couple-Charge-Device (CCD) sensor camera (model AVT Marlin F-145, Allied Vision Technologies, Stadtroda, Alemania) was employed as shown in Figure 3. A macro zoom lens was fitted to the camera to increase the spatial resolution around the crack tip. Thus, a spatial resolution of 14.3 μm per pixel was achieved. DIC processing was performed using the commercial VIC-2D-7 software provided by Correlated Solutions Inc. [28]. For DIC processing, 29 pixel facets were used with a step of one pixel.

The camera system synchronization during recording was carried out by sending a trigger signal commanded by the testing machine controller (model MTS FlexTest 40, MTS Systems Corporation, Eden Prairie, MN, USA) to both cameras.

An additional test was previously conducted to measure the thermoelastic constants for the employed material [29,30,31]. Thus, a plane plate machined from the same material sheet was tested under tensile cyclic load at different values of amplitude and mean stress values. The calibration procedure is detailed the following section.

## 4. Experimental Methodology

### 4.1. Calculating Crack Tip Parameters from TSA and DIC Data

For the calculation of crack tip parameters using full-field information, the Multi-Point Over-Deterministic Method (MPODM) developed by Sanford and Dally [32] was employed to fit both TSA and DIC data to the CJP model stress or displacement equations. Although the mathematical basis (MPODM) is the same for both type of data, each type of data has its own features, and therefore the way in which the data are extracted is different. The applied methodology for each technique is described in the following sections.

#### 4.1.1. Methodology for Fitting Thermoelastic Data

**Figure 4:**
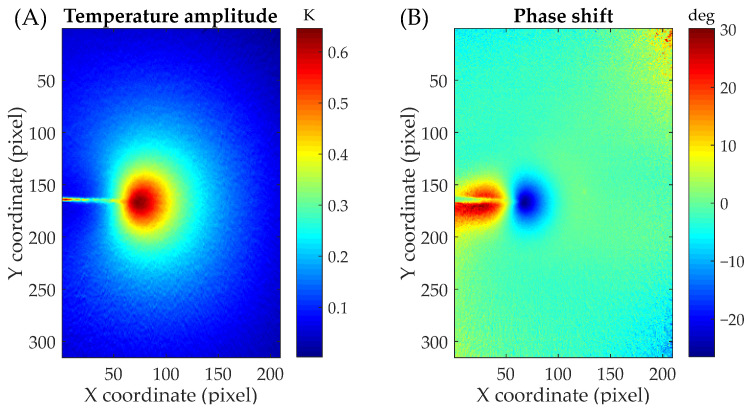
(**A**) Temperature amplitude and (**B**) phase shift maps surrounding the crack tip. Stress ratio of 0.1 and crack length of 8.5 mm.

Once phase and amplitude maps are calculated (see Figure 4), the fitting data is extracted from temperature amplitude maps. Since the CJP model is a linear and elastic model, data should be extracted in the linear and elastic zones and hence out of the crack tip plastic zone. Thus, data is extracted using an annular-shaped mesh that avoids the inclusion of data in the plastic region.

**Figure 5:**
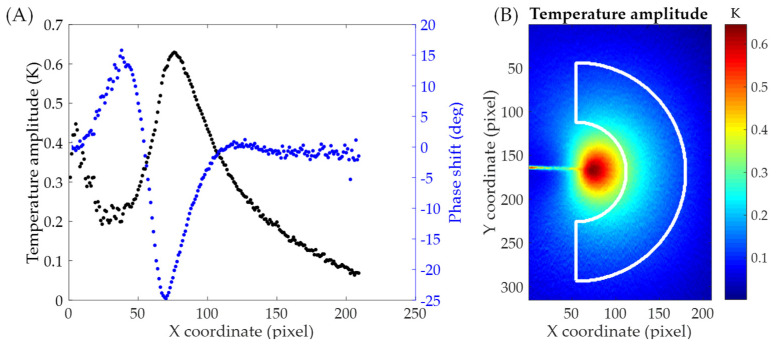
(**A**) Temperature amplitude and phase shift profiles along the crack growth direction to show the different zones surrounding the crack tip for mesh definition and (**B**) annular mesh contour superimposed over the temperature amplitude map.

The mesh geometrical features are obtained by analyzing the different regions in the phase shift (difference between the phases of the load and temperature signals) and temperature maps and profiles along the crack growth direction (see Figure 4 and Figure 5a). Three zones can be primarily identified in the phase shift map (Figure 4b). The green area corresponds to the linear and elastic zone where the phase shift is near zero where adiabatic conditions are achieved. The red and blue zones correspond to the residual plastic wake between the crack flanks and the plastic zone, respectively. In those areas, the phase shift is due to the heat generation because of crack tip plasticity (non-adiabatic conditions). The mesh center should be placed at the crack tip since crack tip field models are referred to the crack tip. In other words, the crack tip is the coordinate origin for any crack tip field model. However, this variable is not overly critical since it is included as fitting unknown, and its value could change during the algorithm. Nevertheless, it is suitable to choose a well-placed value for several reasons. First, the mesh should be well located to avoid the inclusion of plastic data since the other variables are defined from that. Secondly, this value is chosen as an initial guess for solving the nonlinear problem. Therefore, convergence problems due to the initial solution should be avoided if a good value for the crack tip is initially chosen. The crack tip location is identified from the phase shift profile (blue points in Figure 5a) as the zero value between positive and negative shifts (transition between the residual wake and the crack tip plastic zone) [33,34]. The inner radius, which is defined in order to avoid plastic data, is calculated as the difference between the almost zero-phase point in front of the crack tip plastic zone and the previously calculated crack tip. As shown in Figure 5a, the zero-phase point can be clearly identified and obtained from that profile. That point is obtained through nearest neighbor interpolation. Finally, the outer radius, which should be defined in order to capture data within the crack tip singularity zone, is calculated as a crack length fraction (40%) according to Nurse and Patterson’s criterion [35]. However, higher values for that variable do not significantly affect the inferred parameters. Thus, from those parameters, the annular mesh for extracting fitting data is generated as shown in Figure 5b.

Once crack tip temperature data has been extracted, that data is fitted to the thermoelastic equation. In this work, a higher-order thermoelastic equation considering the mean stress effect is employed [18,34] as shown in Equation (12). The stress components in this equation are replaced by the CJP model stress equations (Equations (1)–(3)) to determine the coefficients describing the crack tip fields in the CJP model:

(12) $$\frac{\Delta T}{T_{0}}=\gamma \left({\Delta \sigma }_{x}{+\Delta \sigma }_{y}\right){+\mathrm{bR}}_{f}\left(-2\nu \left({\Delta \sigma }_{x}{\Delta \sigma }_{y}{-\Delta \tau }_{\mathrm{xy}}^{2}\right){+\Delta \sigma }_{x}^{2}{+\Delta \sigma }_{y}^{2}{+2\Delta \tau }_{\mathrm{xy}}^{2}\right),$$

where the Δ symbol indicates the range throughout a loading a cycle, T_0_ is the mean temperature, γ and b are the first- and second-order thermoelastic constants, respectively, and R_f_ is function depending on the stress ratio (R_f_ = (1 − R)/(1 + R)). This nonlinear fitting problem is addressed using an optimization approach that employs an Interior-Point Method [36,37]. The objective function is shown in Equation (13):

(13) $$h\left({\Delta A,\;\Delta B,\;\Delta C,\;\Delta E,\;\Delta F,\;\delta }_{x}{,\;\delta }_{y}\right)={\|\left(\frac{\Delta T}{T_{0}}\right)}^{\exp}-{\left(\frac{\Delta T}{T_{0}}\right)}^{\mathrm{th}}\|,$$

where h is the objective function, δ_x_ and δ_y_ are the crack tip coordinates and superscripts, and exp and th refer to experimental and theoretical array data, respectively. Double vertical bars represent the norm operator. Note from Equation (12) that TSA data directly provides the range of crack tip parameters throughout a loading cycle. The initial solution is constructed by assuming no shielding effect [1] and establishing relationships between the CJP model coefficients and theoretical stress intensity factors and non-singular stresses [18].

#### 4.1.2. Methodology for Fitting Digital Image Correlation Data

**Figure 6:**
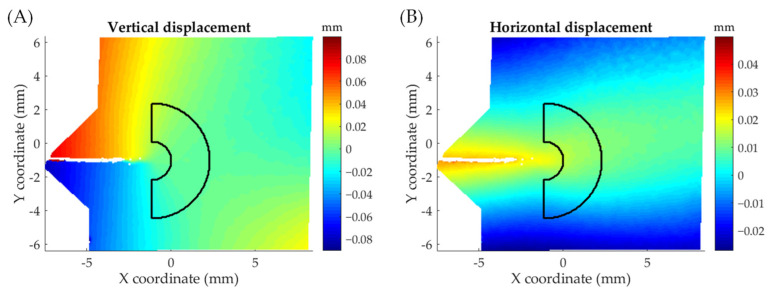
Annular mesh superimposed over DIC displacement maps. (**A**) Vertical displacement map and (**B**) horizontal displacement map. Displacement maps at the maximum load for a crack length of 8.5 mm.

For inferring crack tip parameters from DIC data, the same approach as for thermoelastic data was employed. However, because of the different nature of the data, as TSA measures stresses and DIC displacements, the mesh geometrical features (inner and outer radius and center) were calculated from DIC information. The crack tip position was calculated from vertical displacements using the methodology proposed by Vasco-Olmo et al. [38]. The inner radius was estimated via Dugdale plastic radius approximation [39], which provides a conservative value since overestimating the plastic zone size, and therefore, it ensures that no plastic data is collected. The outer radius was defined employing the same criterion as for thermoelastic data (40% of the crack length). Figure 6 shows the generated mesh superimposed over vertical (Figure 6a) and horizontal displacement maps (Figure 6b).

For fitting any crack tip field model to DIC data, three additional variables modelling a small rigid body motion should be included. Thus, the objective function leads as shown in Equation (14);

(14) $$g\left({A,\;B,\;C,\;E,F,\;u}_{x0}{,u}_{y0}{,R}_{\mathrm{xy}}{,\delta }_{x}{,\delta }_{y}\right)=\|U^{\mathrm{th}}{-U}^{\exp}\|,$$

where u_x0_, u_y0_, and R_xy_ are the coefficients modelling a small-displacement rigid body motion (displacements along x and y directions and an in-plane rotation), and U is an array containing both vertical (u_y_) and horizontal (u_x_) displacements. To solve that optimization problem, the same approach and algorithm as for TSA data were employed. The maximum and minimum load displacement fields were analyzed to calculate crack tip parameter ranges from DIC data.

### 4.2. Determination of the Thermal Calibration Contants

As previously introduced, a calibration test [29,30,31,34] was performed to obtain the thermal calibration constants for the employed material. A plane plate subjected to cyclic uniaxial loading allows a uniaxial stress state to be obtained. Each stress state simplifies the thermoelastic equation and allows the thermoelastic constants to be obtained simply. The simplified thermoelastic equation for a uniaxial case is given by the following equation:

(15) $$\frac{\Delta T}{T_{0}\Delta \sigma }{=\gamma +b\sigma }_{m},$$

where σ_m_ is the mean stress. Note that for the uniaxial case of Equation (12), the product of R_f_ and Δσ gives σ_m_. Thus, by fitting Equation (15) (line equation) to temperature data for different values of mean and alternant stress, the thermoelastic constants can be determined. The different stress states used for the calibration are displayed in Table 3.

Finally, the obtained values for the thermoelastic constants were 3.1765 × 10^−6^ MPa^−1^ and 4.3715 × 10^−9^ MPa^−2^ for the first- and second-order constants, respectively.

## 5. Results and Discussion

**Figure 7:**
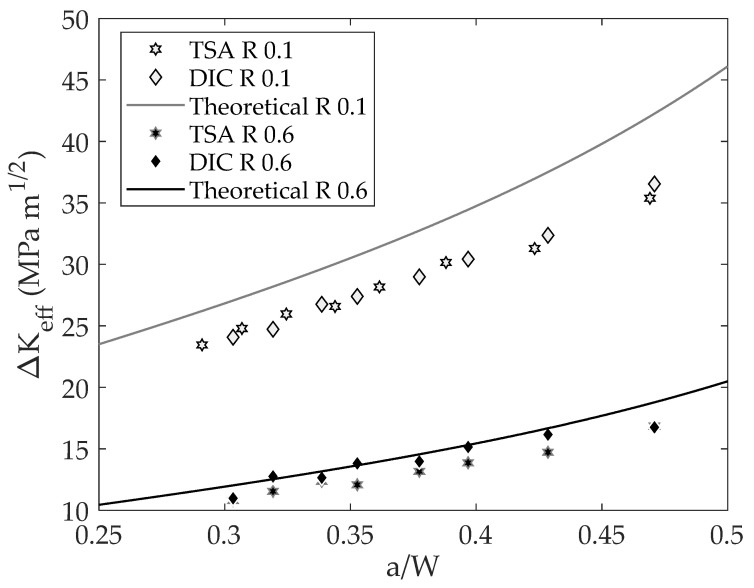
CJP model effective stress intensity factor range vs. the dimensionless crack length.

**Figure 8:**
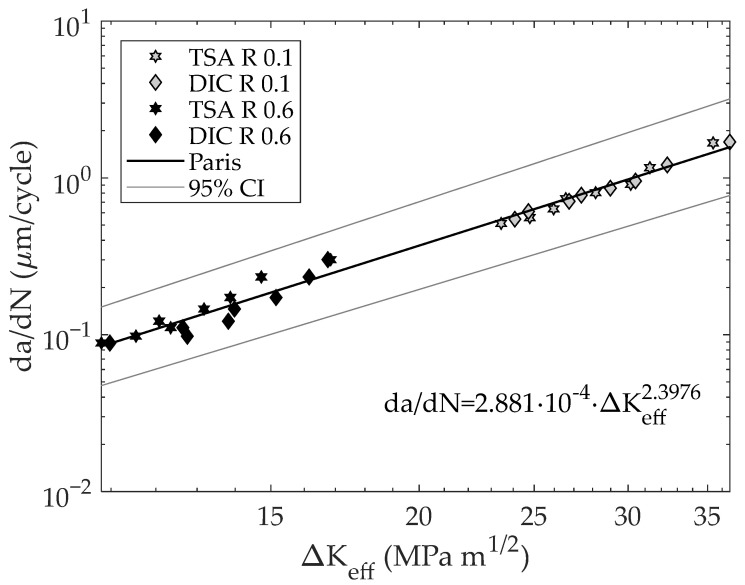
Log–log da/dN vs ΔK_eff_ plot along with the fitted Paris curve and the fitting 95% confidence lines. Fitting R-square coefficient of 98.53%. 95% CI indicates the 95% confidence intervals.

Figure 7 shows the experimentally obtained ΔK_eff_ from both TSA and DIC data along with the theoretical ΔK [40] for the different tested stress ratios versus the dimensionless crack length. As a first point, it can be seen how the calculated SIF ranges from DIC and TSA agree well for both stress ratios. For the high stress ratio, slight differences between TSA and DIC data were found; however, these small differences may be fairly attributed to the inherent noise of the experimental measurement since for small stress amplitudes (high stress ratios) the noise could be significantly higher than for high stress amplitudes (low stress ratio). As shown in Figure 6, for both stress ratios the effective SIF ranges are lower than the nominal but more significantly for the lower stress ratio. This fact seems meaningful as a higher closure influence is expected for a low stress ratio than for a high stress ratio. Figure 8 shows a log–log da/dN − ΔK_eff_ plot. As shown here, the effective CJP model stress intensity factor range correlates well with fatigue crack growth rates for both stress ratios. In other words, both data (0.1 and 0.6 stress ratios) are in the same line, and no offset between stress ratios was found. In Figure 8, the Paris equation fitted to those data is also displayed. The obtained coefficients were 2.8113 × 10^−4^ for the proportional coefficient and 2.3976 for the potential law exponent (crack growth rates in μm/cycle and SIFs ranges in MPa m^1/2^), which agree well with those obtained by Yang et al. [25] for the tested material (1.7636 × 10^−4^ for the proportional coefficient and 2.4146 for the potential law exponent). In addition, all the data keeps within the 95% fitting confidence bounds as shown in Figure 8. Regarding the fitting quality, a value of 98.53% in terms of the R-square parameter was obtained. The fitting of da/dN − ΔK data to Paris’s equation was performed using Linear Least Squares for a line equation by first linearizing the power law through logarithms. Thus, possible errors because of the use of numerical methods were avoided.

Above, the CJP model ΔK_eff_ has been verified as a suitable crack tip governing, which provides an effective rationalization of fatigue crack growth rates. Next, this parameter is analyzed by trying to separate into two SIF ranges, one of them representing the linear and elastic component and another one that characterizes the crack-shielding influence. For convenience, ΔK_eff_ (Equation (9)) is decomposed into terms called Γ_1_ and Γ_2_ as displayed in Equations (16) and (17), respectively. As coefficient E is related to crack-shielding influence, the term Γ_1_ should corresponds to the linear and elastic components, and Γ_2_ to the retardation component:

(16) $$\Gamma_{1}=\sqrt{\frac{\pi }{2}}\left(\Delta A-3\Delta B\right),$$

(17) $$\Gamma_{2}=\sqrt{\frac{\pi }{2}}\left(4.56\Delta E\right),$$

**Figure 9:**
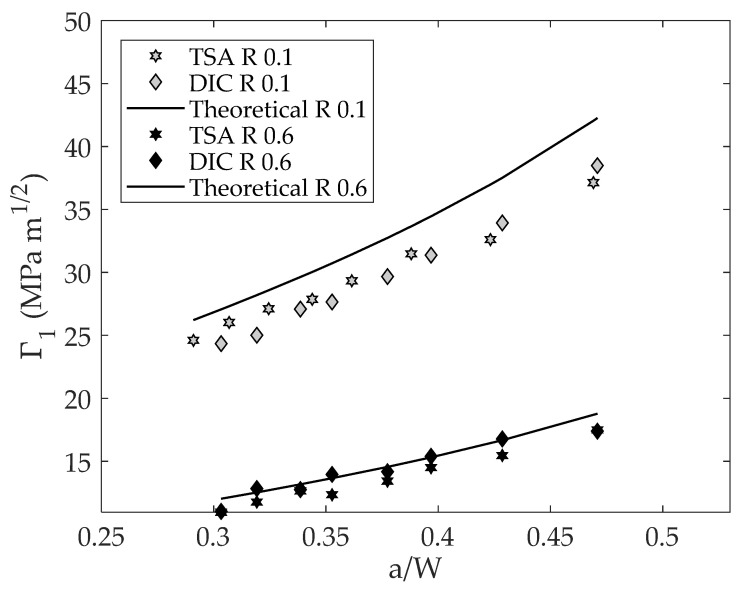
Linear and elastic components of the CJP model effective stress intensity factor vs. the dimensionless crack length.

Note that the sum of Equations (15) and (16) gives ΔK_eff_. Figure 9 shows the parameter Γ_1_ versus the dimensionless crack length. As shown in Figure 9, a good agreement was also found between DIC and TSA data for both stress ratios, and the values of parameter Γ_1_ also differ from the theoretical value being more significant for the low stress ratio than for the high stress ratio. The reduction compared to the nominal that occurred at the low stress ratio may be attributed to the premature contact between crack flanks causing crack closure that can affect both the measured temperature signals and displacement fields. That is, for TSA data, the measured temperature signal could be attenuated due to the premature contact between crack flanks [17], and for DIC data, the same phenomenon could occur for displacements fields [15].

**Figure 10:**
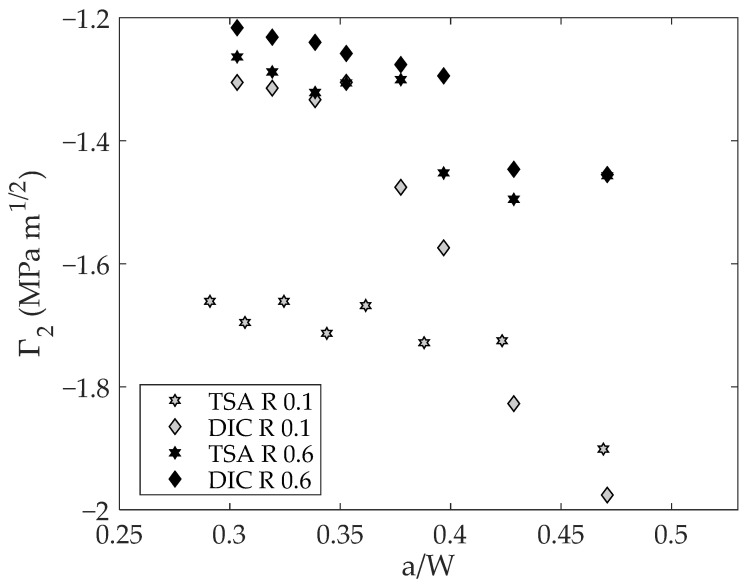
Shielding component of the CJP model effective stress intensity factor range.

Figure 10 shows parameter Γ_2_, which is related to the crack shielding. The trend of this parameter is increasing as the crack grows, which is meaningful since the shielding effect is expected to increase as the crack grows at constant amplitude loading therefore increasing the size of the crack tip plastic zone. The differences at small crack lengths for the 0.1 stress ratio might be due to inherent experimental noise, which might affect this variable since its magnitude order is around 0.5 MPa m^1/2^.

**Figure 11:**
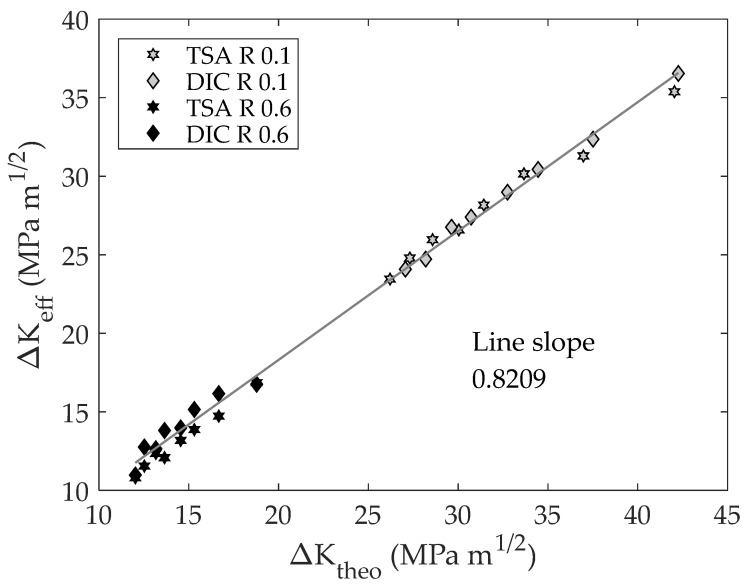
CJP model effective stress intensity factor range vs. theoretical stress intensity factor range.

**Figure 12:**
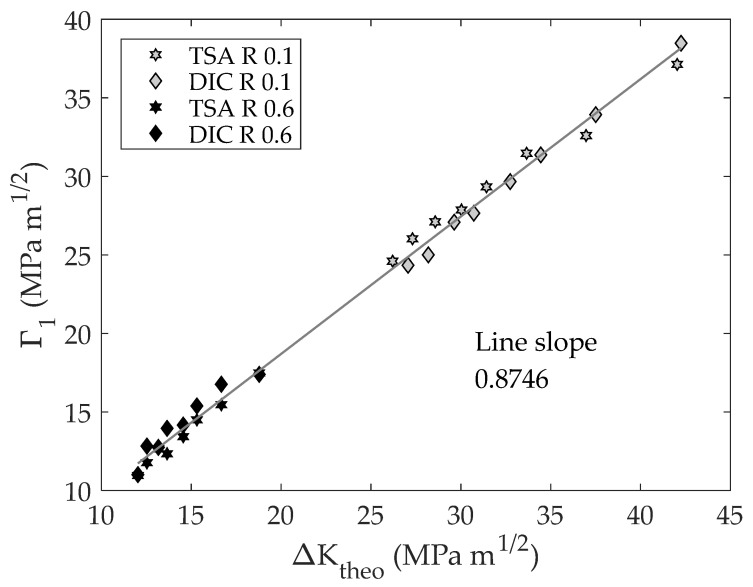
Elastic term of the CJP model effective stress intensity factor range vs. theoretical stress intensity factor range.

Finally, to quantify differences between the CJP model effective stress intensity factor and its linear and elastic terms, both parameters are analyzed by comparing them with the theoretical values as shown in Figure 11 and Figure 12. This representation allows the difference between each parameter and the theoretical value to be assessed without dimensions. Thus, if a line is fitted, as shown in Figure 11 and Figure 12, the slope corresponds to the average ratio between each parameter and the theoretical value.

As shown in Figure 11 and Figure 12, a linear relationship between both parameters and the theoretical SIF was found. This fact means that there is proportionality between both parameters, and, hence, those ratios are constant for different crack lengths. The fitted slopes are 0.8209 and 0.8746 for the effective stress intensity factor and the elastic component, respectively. These results along with Figure 8 demonstrate the importance of considering the plasticity influence (crack shielding) beyond the crack closure effect on modelling fatigue crack growth rates.

## 6. Conclusions

In this work, the crack-shielding effect on fatigue crack growth has been studied in pure grade 2 titanium CT specimens by combining the CJP model along with TSA and DIC data. From the above-reported results, the following conclusions can be established:

- There is convergence between the obtained SIF ranges from TSA and DIC data (mean deviation between data of around 2%) indicating the validity of the obtained results.
- The CJP model effective stress intensity factor range provides an effective rationalization of fatigue crack growth rates since those values fall along the same power law when ΔK_eff_ is used as the fatigue crack driving force.
- The CJP model is able to quantify both the elastic and the retardation components of the stress intensity existing at the tip of a fatigue crack.
- The CJP model also demonstrates that crack closure is not the only factor involved in crack growth retardation, and it is important to consider the effects of the crack tip plasticity during fatigue crack growth (crack shielding), which can reduce the value of the effective SIF up to 7%, to a proper modelling of fatigue crack growth rates using Paris’s law.
- Further study might consider a recent and novel formulation of the CJP model also including the crack-blunting effect [41].

## Figures and Tables

**Table 1:** Chemical composition (%wt) of pure grade 2 titanium [25].

Fe	C	N	O	H	Ti
0.1	0.01	>0.01	0.12	0.002	Balance

**Table 2:** Mechanical properties of pure grade 2 titanium [25].

Young’s Modulus (GPa)	0.2% Yield Stress (MPa)	Ultimate Tensile Strength (MPa)	Elongation at Failure (%)	Poisson’s Ratio
105	390	448	20	0.34

**Table 3:** Loading cases used for the thermal calibration.

Amplitude (N)	Mean Component (N)
700	1400
700	1600
700	1800
1000	1400
1000	1600
1000	1800
1200	1400
1200	1600
1200	1800

## Data Availability

The data that support the findings of this study are available from the corresponding author upon reasonable request.
